# Effects of Convective Heat and Mass Transfer in Flow of Powell-Eyring Fluid Past an Exponentially Stretching Sheet

**DOI:** 10.1371/journal.pone.0133831

**Published:** 2015-09-01

**Authors:** T. Hayat, Yusra Saeed, A. Alsaedi, Sadia Asad

**Affiliations:** 1 Department of Mathematics, Quaid-i-Azam University 45320, Islamabad 44000, Pakistan; 2 Nonlinear Analysis and Applied Mathematics (NAAM) Research Group, Department of Mathematics, Faculty of Science, King Abdulaziz University, Jeddah 21589, Saudi Arabia; China University of Mining and Technology, CHINA

## Abstract

The aim here is to investigate the effects of convective heat and mass transfer in the flow of Eyring-Powell fluid past an inclined exponential stretching surface. Mathematical formulation and analysis have been performed in the presence of Soret, Dufour and thermal radiation effects. The governing partial differential equations corresponding to the momentum, energy and concentration are reduced to a set of non-linear ordinary differential equations. Resulting nonlinear system is computed for the series solutions. Interval of convergence is determined. Physical interpretation is seen for the embedded parameters of interest. Skin friction coefficient, local Nusselt number and local Sherwood number are numerically computed and examined.

## Introduction

The flows of non-Newtonian fluids over a stretching surface with heat transfer have many applications in engineering processes like polymers extrusion, paper production, food processing, glass fiber, drawing of plastic films, slurry transporting and many others. Crane [1] initiated the pioneering work for closed form solution of viscous flow over a linear stretching surface. Afterwards a large amount of research work has been reported in this direction through different aspects of suction/blowing, heat and mass transfer, different non-Newtonian models, magnetohydrodynamics, different stretching velocities of surface etc. In particular, the combined influence of heat and mass transfer is important in several engineering applications including metallurgy, solar collectors, combustion systems, chemical engineering, nuclear reactor safety etc. Such transport processes are governed by the buoyancy forces from both thermal and mass diffusion in heating and cooling chambers, energy processes, space technology, solar power technology etc. Inspired by such facts, various researchers are still engaged for the discussion of heat and mass transfer effects in flow over a stretching surface with radiation effect (see [2–10]). It is also noted that heat and mass transfer in these studies and many others have been discussed by prescribing both the constant temperature and concentration or by constant heat and mass fluxes at the stretching surface. Recently some contributions have been made to discuss the heat transfer mechanism in such flow with convective temperature condition at the surface (see [11–20]).

In present communication, we address the convective heat and mass transfer conditions in the radiative flow of Powell-Eyring fluid past an inclined exponentially stretching surface. Soret and Dufour effects are taken into account. The considered Powell-Eyring fluid model is although mathematically complex but it has certain advantages over the other non-Newtonian fluid models. Firstly, it is deduced from kinetic theory of liquid rather than the empirical relation. Secondly, it correctly reduces to Newtonian behavior for low and high shear rates. Here suitable transformations are utilized to convert the governing partial differential equations into the ordinary differential equations. Convergent series solutions of the problems are accomplished by using homotopy analysis method (HAM [21–30]). This method is capable of solving a wide range of nonlinear problems, particularly when the nonlinearity is strong. The origin of homotopy lies in topology. Two mathematical objects are said to be homotopic if one can be continuously deformed into the other. Homotopy is widely applied in numerical techniques. The HAM here is preferred through the reasons as follows. Unlike perturbation techniques the homotopy analysis method is independent of small/large parameter. It itself can provide us with a convenient way to adjust and control the convergence region and rate of approximation series when necessary. Interesting physical quantities are analyzed through plots and numerical values.

### Mathematical Formulation

**Fig 1:**
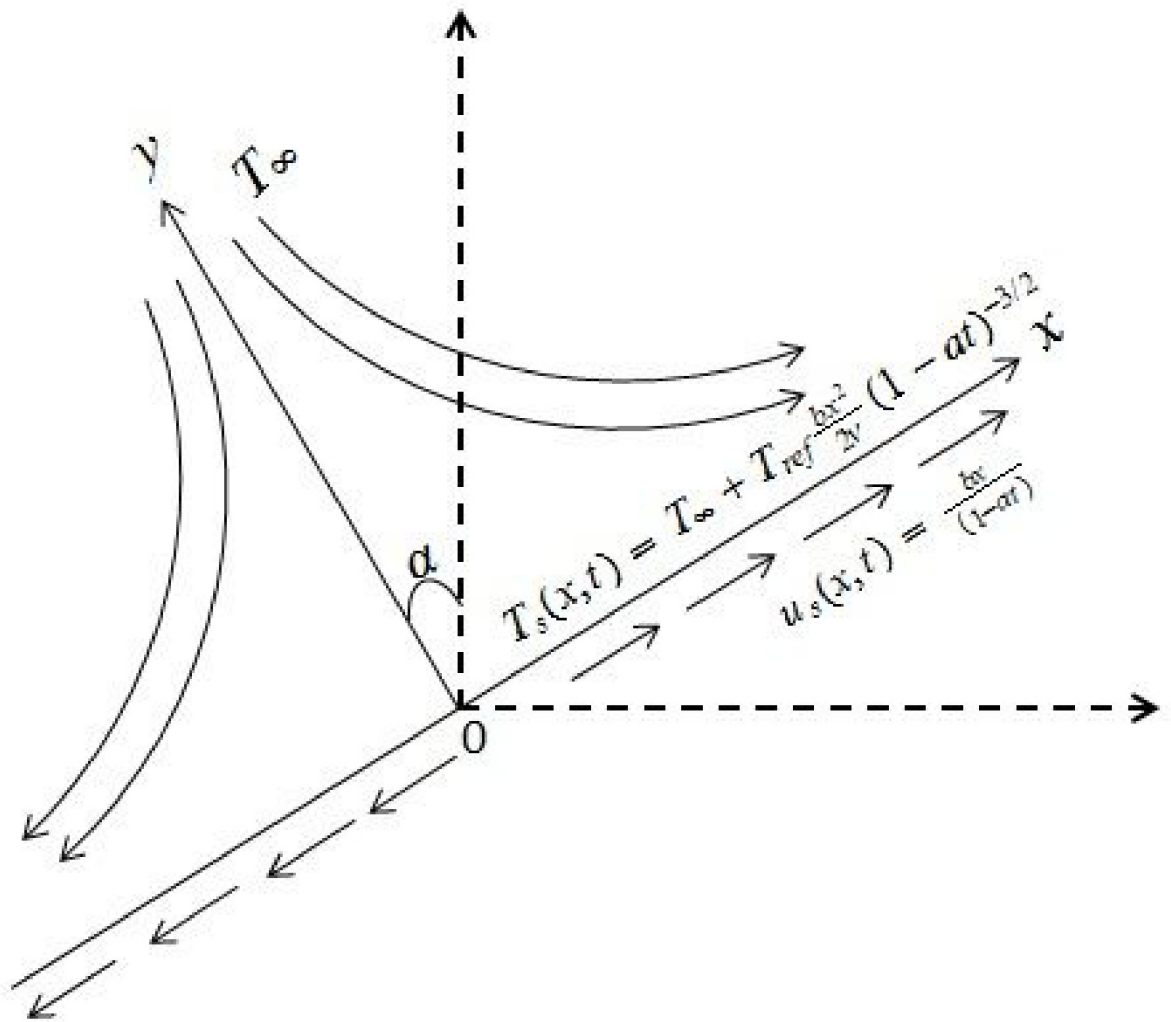
Physical model and coordinate system.

We consider steady two-dimensional flow of an incompressible Powell-Eyring fluid past an exponential stretching sheet. Simultaneous effects of heat and mass transfer are considered. The sheet is inclined through angle *α*. Both conditions of heat and mass transfer at the surface are of convective type (see Fig 1). Under the usual boundary layer and Rosseland approximations, the present flow problem is governed by the following equations.

$$\frac{\partial u}{\partial x}+\frac{\partial v}{\partial y}=0,$$ (1)

u∂u∂x+v∂u∂y=(ν+1ρbc)∂2u∂y2−12bc3ρ[(∂u∂y)2∂2u∂y2]+gβ(T−T∞)cos(α)+gβc(C−C∞)cos(α), (2)

$$u\frac{\partial T}{\partial x}+v\frac{\partial T}{\partial y}=\alpha *\frac{\partial^{2}T}{\partial y^{2}}-\frac{1}{\rho c_{p}}\frac{\partial q_{r}}{\partial y}+\frac{D_{m}k_{T}}{c_{p}c_{s}}\frac{\partial^{2}C}{\partial y^{2}},$$ (3)

$$u\frac{\partial C}{\partial x}+v\frac{\partial C}{\partial y}=D_{m}\frac{\partial^{2}C}{\partial y^{2}}+\frac{D_{m}k_{T}}{T_{m}}\frac{\partial^{2}T}{\partial y^{2}},$$ (4)

$$u=U_{w}\left(x\right),\;v=0,\;-k\frac{\partial T}{\partial y}=h\left(T_{f}-T\right),\;-D_{m}\frac{\partial C}{\partial y}=k_{m}\left(C_{f}-C\right)\;\text{at}\;y=0,$$

$$u\to 0,\;T\to T_{\infty },\;C\to C_{\infty }\;\text{as}\;y\to \infty ,$$ (5)

where u and v represent the velocity components along α the x and y directions respectively, *U*_*w*_(*x*) = *U*_0_ *e* ^*x* / *l*^ is the stretching velocity of sheet, *U*_0_ is the reference velocity, l is the reference length, b and c are the material fluid parameters, *ρ* is the density, *ν* is the kinematic viscosity, g is the acceleration due to gravity, *β* is the volumetric coefficient of thermal expansion, *β*_*c*_ is the concentration expansion, T is the fluid temperature, *T*_∞_ is the ambient temperature, *C* is the fluid concentration, *C*_∞_ is the ambient concentration, α* is the thermal diffusivity, k is the thermal conductivity, *c*_*p*_ is the specific heat, $q_{r}=-\frac{16\sigma *T_{\infty }^{3}}{3k*}\frac{\partial T}{\partial y}$ is the radiative heat flux, k* is the mean absorption coefficient, *σ* is the Stefan-Boltzmann constant, *c*_*s*_ is the concentration susceptibility, *D*_*m*_ is the molecular diffusivity of the species concentration, *k*_*T*_ is the thermal diffusion ratio, h is the wall heat transfer coefficient, *k*_*m*_ is the wall mass transfer coefficient, *T*_*m*_ is the mean fluid temperature, convective heating process is characterized by temperature *T*_*f*_ and associated concentration near the surface is *C*_*f*_.

We introduce the following dimensionless variables

$$\begin{array}{l} \;u=U_{0}e^{x/l}f\prime (\eta ),\;v=-\sqrt{\frac{\nu U_{0}}{2l}}e^{x/2l}\left[f(\eta )+\eta f\prime (\eta )\right],\\ \theta (\eta )=\frac{T-T_{\infty }}{T_{f}-T_{\infty }},\;\phi \left(\eta \right)=\frac{C-C_{\infty }}{C_{f}-C_{\infty }},\;\eta =\sqrt{\frac{U_{0}}{2\nu l}}e^{x/2l}y. \end{array}$$ (6)

With the help of above dimensionless variables, Eq (1) is identically satisfied and Eqs (2–5) yield

$$\left(1+\lambda_{1}\right)f\prime \prime \prime +ff\prime \prime -2f^{\prime^{2}}-\lambda_{2}\lambda_{1}f^{\prime \prime^{2}}f\prime \prime \prime +\lambda \theta \cos\left(\alpha \right)+\delta \phi \cos\left(\alpha \right)=0,$$ (7)

$$\left(1+\frac{4}{3}R\right)\theta \prime \prime +\mathrm{Pr}\;f\theta \prime +\mathrm{Pr}\;Du\phi \prime \prime =0,$$ (8)

ϕ″+ScSrθ″+Scfϕ′=0. (9)

$$f=0,\;f\prime =1,\;\theta \prime \left(0\right)=-Bi_{1}\left(1-\theta \left(0\right)\right),\;\phi \prime \left(0\right)=-Bi_{2}\left(1-\phi \left(0\right)\right)\;\text{at}\;\eta =0,$$

f′=0,θ=0,ϕ=0atη=∞, (10)

where *λ*_1_ and *λ*_2_ are the fluid parameters, *λ* denotes thermal buoyancy parameter, *δ* stands for solutal buoyancy parameter, Pr is the Prandtl number, *Du* is the Dufour number, *Sr* is the Soret number, *Sc* is the Schmidt number, *Bi*_1_ is the thermal Biot number, *Bi*_2_ is the concentration Biot number and *R* is the radiation parameter. The definitions of these parameters are

$$\begin{array}{l} \;\lambda_{2}=\frac{\rho U_{0}^{3}e^{3x/l}}{4\mu c^{2}l},\;Bi_{1}=\frac{h}{k}\sqrt{\frac{2\upsilon l}{U_{0}}}\frac{1}{e^{2x/l}},\;Du=\frac{D_{m}k_{T}(C_{f}-C_{\infty })}{\nu c_{\rho }c_{s}(T_{f}-T_{\infty })},\;\lambda_{1}=\frac{1}{\mu bc},\\ Bi_{2}=\frac{k_{m}}{D_{m}}\sqrt{\frac{2\upsilon l}{U_{0}}}\frac{1}{e^{2x/l}},\;Sr=\frac{D_{m}k_{T}(T_{f}-T_{\infty })}{\nu T_{m}(C_{f}-C_{\infty })},\;R=\frac{4\sigma T_{\infty }^{3}}{kk*},\;\lambda =\frac{g\beta \left(T_{f}-T_{\infty }\right)l^{2}}{xU_{0}^{2}},\\ \;Sc=\frac{\nu }{D_{m}}.\mathrm{Pr}=\frac{\nu }{\alpha *},\;\delta =\frac{g\beta_{c}\left(C_{f}-C_{\infty }\right)l^{2}}{xU_{0}^{2}}. \end{array}$$ (11)

The local Nusselt number *Nu*_*x*_, local Sherwood number *Sh*_*x*_ and skin-friction coefficient $C_{f_{x}}$ are defined by

$$Nu_{x}=\frac{xq_{w}}{k(T_{f}-T_{\infty })};\;q_{w}={-k\frac{\partial T}{\partial y}-\frac{16\sigma T_{\infty }^{3}}{3k*}\frac{\partial T}{\partial y}|}_{y=0}.$$ (12)

$$Sh_{x}=\frac{J_{w}x}{D_{m}\left(C_{f}-C_{\infty }\right)};\;J_{w}={-D_{m}\frac{\partial C}{\partial y}|}_{y=0}.$$ (13)

$$C_{f_{x}}=\frac{2\tau_{w}}{\rho U_{w}^{2}};\;\tau_{w}={\left(\mu +\frac{1}{bc}\right)\frac{\partial u}{\partial y}-\frac{1}{6b}{\left(\frac{1}{c}\frac{\partial u}{\partial y}\right)}^{3}|}_{y=0}.$$ (14)

Dimensionless forms of Eqs (12–14) are:

Nuxx/2lRex=−(1+43R)θ′(0). (15)

Shxx/2lRex=−ϕ′(0). (16)

CfxRex2x/2l=[(1+λ1)f″(0)−λ1λ23f′′3(0)], (17)

where Rex=Uw(x)xυ is the local Reynolds number.

### Methodology of Solution

It should be noted that there is a great freedom to choose initial guess and auxiliary linear operator. Also there are some fundamental rules which direct us to choose the mentioned parameters in more efficient way. Therefore, initial guesses for the velocity, temperature and concentration fields are taken in such a way that they satisfy the boundary conditions given in Eq (10). We choose linear operators involving base functions of the exponential type. In fact such preferences of exponential type function accelerate the convergence of the series solutions.

$$f_{0}(\eta )=1-e^{-\eta },\;\theta_{0}(\eta )=\frac{Bi_{1}}{\left(Bi_{1}+1\right)}e^{-\eta },\;\phi_{0}(\eta )=\frac{Bi_{2}}{\left(Bi_{2}+1\right)}e^{-\eta },$$ (18)

$$L_{f}=f\prime \prime \prime -f\prime ,\;L_{\theta }=\theta \prime \prime -\theta ,\;L_{\theta }=\phi \prime \prime -\phi ,$$ (19)

subject to the properties

$$L_{f}(C_{1}+C_{2}e^{\eta }+C_{3}e^{-\eta })=0,$$ (20)

$$L_{\theta }(C_{4}e^{\eta }+C_{5}e^{-\eta })=0,$$ (21)

$$L_{\phi }(C_{6}e^{\eta }+C_{7}e^{-\eta })=0,$$ (22)

where *C*_*i*_ (i = 1–7) are the arbitrary constants determined from the boundary conditions. If p ∈[0,1] denotes an embedding parameter, $\hbar_{f}$, $\hbar_{\theta }$ and $\hbar_{\phi }$ the non-zero auxiliary parameters then the zeroth order deformation problems are

$$\left(1-p\right)L_{f}\left[\hat{f}(\eta ;p)-f_{0}(\eta )\right]=ph_{f}N_{f}\left[\hat{f}(\eta ;p),\hat{\theta }(\eta ;p),\hat{\phi }(\eta ;p)\right],$$ (23)

$$\left(1-p\right)L_{\theta }\left[\hat{\theta }(\eta ;p)-\theta_{0}(\eta )\right]=ph_{\theta }N_{\theta }\left[\hat{f}(\eta ;p),\hat{\theta }(\eta ;p),\hat{\phi }(\eta ;p)\right],$$ (24)

$$\left(1-p\right)L_{\phi }\left[\hat{\phi }(\eta ;p)-\phi_{0}(\eta )\right]=ph_{\phi }N_{\phi }\left[\hat{f}(\eta ;p),\hat{\theta }(\eta ;p),\hat{\phi }(\eta ;p)\right],$$ (25)

$$\hat{f}(0;p)=0,\;\hat{f}\prime (0;p)=1,\;\hat{f}\prime (\infty ;p)=0,\;\hat{\theta }\prime (0;p)=-Bi_{1}\left[1-\hat{\theta }(0;p))\right],$$

$$\hat{\phi }\prime (0;p)=-Bi_{2}\left[1-\hat{\phi }(0;p)\right],\;\hat{\theta }(\infty ;p)=0,\;\hat{\phi }(\infty ;p)=0,$$ (26)

where **N**_*f*_, **N**_*θ*_ and **N**_*ϕ*_ are the nonlinear operators defined as follows:

Nf[f^(η;p),θ^(η;p),ϕ^(η;p)]=(1+λ1)∂3f^(η,p)∂η3+f^(η,p)∂2f^(η,p)∂η2−2(∂f^(η,p)∂η)2−λ2λ1(∂2f^(η,p)∂η2)2∂3f^(η,p)∂η3+λθ^(η,p)cos(α)+δϕ^(η,p)cos(α), (27)

$$N_{\theta }[\hat{\theta }(\eta ;p),\hat{f}(\eta ;p),\hat{\phi }(\eta ;p)]=\left(1+\frac{4}{3}R\right)\frac{\partial^{2}\hat{\theta }(\eta ,p)}{\partial \eta^{2}}+\mathrm{Pr}\hat{f}(\eta ,p)\frac{\partial \hat{\theta }(\eta ,p)}{\partial \eta }+\mathrm{Pr}Du\frac{\partial^{2}\hat{\phi }(\eta ,p)}{\partial \eta^{2}},$$ (28)

$$N_{\phi }[\hat{\phi }(\eta ;p),\hat{\theta }(\eta ;p),\hat{f}(\eta ;p)]=\frac{\partial^{2}\hat{\phi }(\eta ,p)}{\partial \eta^{2}}+Sc\hat{f}(\eta ,p)\frac{\partial \hat{\phi }(\eta ,p)}{\partial \eta }+ScSr\frac{\partial^{2}\hat{\theta }(\eta ,p)}{\partial \eta^{2}}.$$ (29)

For p = 0 and p = 1 we have

$$\hat{f}(\eta ;0)=f_{0}(\eta ),\hat{\theta }(\eta ;0)=\theta_{0}(\eta ),\hat{\phi }(\eta ;0)=\phi_{0}(\eta ),$$

$$\hat{f}(\eta ;1)=f(\eta ),\;\hat{\theta }(\eta ;1)=\theta (\eta ),\;\hat{\phi }(\eta ;1)=\phi (\eta ),$$ (30)

and when p variation is taken from 0 to 1 then *f*(*η*,*p*), *θ*(*η*,*p*) and *ϕ*(*η*,*p*) approach *f*_0_(*η*), *θ*_0_(*η*) and *ϕ*_0_(*η*) to *f*(*η*), *θ*(*η*) and *ϕ*(*η*). Now *f*, *θ* and *ϕ* in Taylor's series can be expanded as follows:

$$f(\eta ,p)=f_{0}(\eta )+\sum_{m=1}^{\infty }f_{m}(\eta )p^{m}.$$ (31)

$$\theta (\eta ,p)=\theta_{0}(\eta )+\sum_{m=1}^{\infty }\theta_{m}(\eta )p^{m}.$$ (32)

$$\phi (\eta ,p)=\phi_{0}(\eta )+\sum_{m=1}^{\infty }\phi_{m}(\eta )p^{m}.$$ (33)

$$f_{m}(\eta )=\frac{1}{m!}{\frac{\partial^{m}f(\eta ;p)}{\partial \eta^{m}}|}_{p=0},\;\theta_{m}(\eta )=\frac{1}{m!}{\frac{\partial^{m}\theta (\eta ;p)}{\partial \eta^{m}}|}_{p=0},\;\phi_{m}(\eta )=\frac{1}{m!}{\frac{\partial^{m}\phi (\eta ;p)}{\partial \eta^{m}}|}_{p=0}.$$ (34)

Here the convergence depends upon $\hbar_{f}$, $\hbar_{\theta }$ and $\hbar_{\phi }$. By proper choices of $\hbar_{f}$, $\hbar_{\theta }$ and $\hbar_{\phi }$, the series (31–33) converge for p = 1 and hence

$$f(\eta )=f_{0}(\eta )+\sum_{m=1}^{\infty }f_{m}(\eta ).$$ (35)

$$\theta (\eta )=\theta_{0}(\eta )+\sum_{m=1}^{\infty }\theta_{m}(\eta ).$$ (36)

$$\phi (\eta )=\phi_{0}(\eta )+\sum_{m=1}^{\infty }\phi_{m}(\eta ).$$ (37)

The *m* ^*th*^- order deformation problems are

$$L_{f}[f_{m}(\eta )-\chi_{m}f_{m-1}(\eta )]=\hbar_{f}R_{f}^{m}(\eta ).$$ (38)

$$L_{\theta }[\theta_{m}(\eta )-\chi_{m}\theta_{m-1}(\eta )]=\hbar_{\theta }R_{\theta }^{m}(\eta ).$$ (39)

$$L_{\phi }[\phi_{m}(\eta )-\chi_{m}\phi_{m-1}(\eta )]=\hbar_{\phi }R_{\phi }^{m}(\eta ).$$ (40)

$$f_{m}(0)=f_{m}^{\prime }(0)=f_{m}^{\prime }(\infty )=0,\;\theta_{m}^{\prime }(0)-Bi_{1}\theta_{m}(0)=\theta_{m}(\infty )=0,$$

$$\phi_{m}^{\prime }(0)-Bi_{2}\phi_{m}(0)=\phi_{m}(\infty )=0.$$ (41)

Rfm(η)=(1+λ1)fm−1′′′+∑k=0m−1(fm−1−kfk′′−2fm−1−k′fk′)−λ2λ1∑k=0m(∑l=0kfl′′fk−l′′)fm−k′′′+(λθm−1+δϕm−1)cos(α). (42)

$$R_{\theta }^{m}(\eta )=\left(1+\frac{4}{3}R\right)\theta_{m-1}^{\prime \prime }+\mathrm{Pr}\left(\sum_{k=0}^{m-1}\theta_{m-1-k}^{\prime }f_{k}\right)+\mathrm{Pr}Du\phi_{m-1}^{\prime \prime }.$$

$$R_{\phi }^{m}(\eta )=\phi_{m-1}^{\prime \prime }+ScSr\theta_{m-1}^{\prime \prime }+Sc\left(\sum_{k=0}^{m-1}\phi_{m-1-k}^{\prime }f_{k}\right).$$ (43)

$$\chi_{m}=\{\begin{array}{c} 0,\;m\leq 1\\ 1,\;m>1 \end{array}$$ (44)

The general solutions of Eqs (38–41) are given by

$$f_{m}(\eta )=f_{m}^{*}(\eta )+C_{1}+C_{2}e^{\eta }+C_{3}e^{-\eta },$$ (45)

$$\theta_{m}(\eta )=\theta_{m}^{*}(\eta )+C_{4}e^{\eta }+C_{5}e^{-\eta },$$ (46)

$$\phi_{m}(\eta )=\phi_{m}^{*}(\eta )+C_{6}e^{\eta }+C_{7}e^{-\eta },$$ (47)

where $f_{m}^{*}$, $\theta_{m}^{*}$ and $\phi_{m}^{*}$ are the particular solutions. Constants *C*_*i*_ (*i* = 1–7) are determined by boundary conditions (40).

### Convergence of the HAM Solution

**Fig 2:**
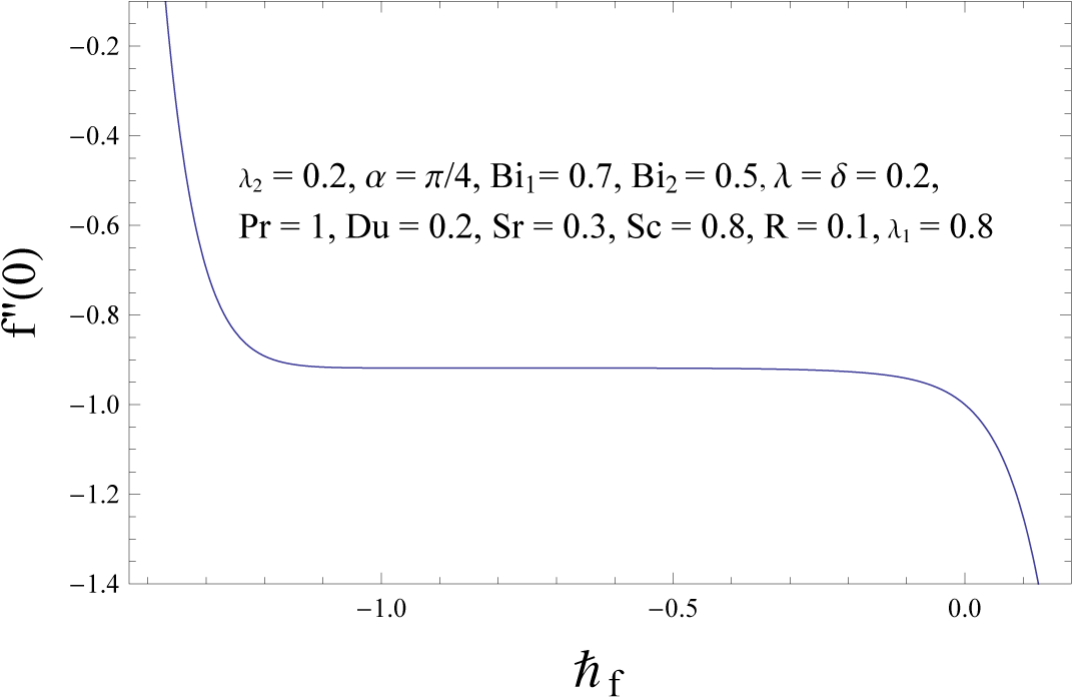
$\hbar_{f}$ – curve for velocity field.

**Fig 3:**
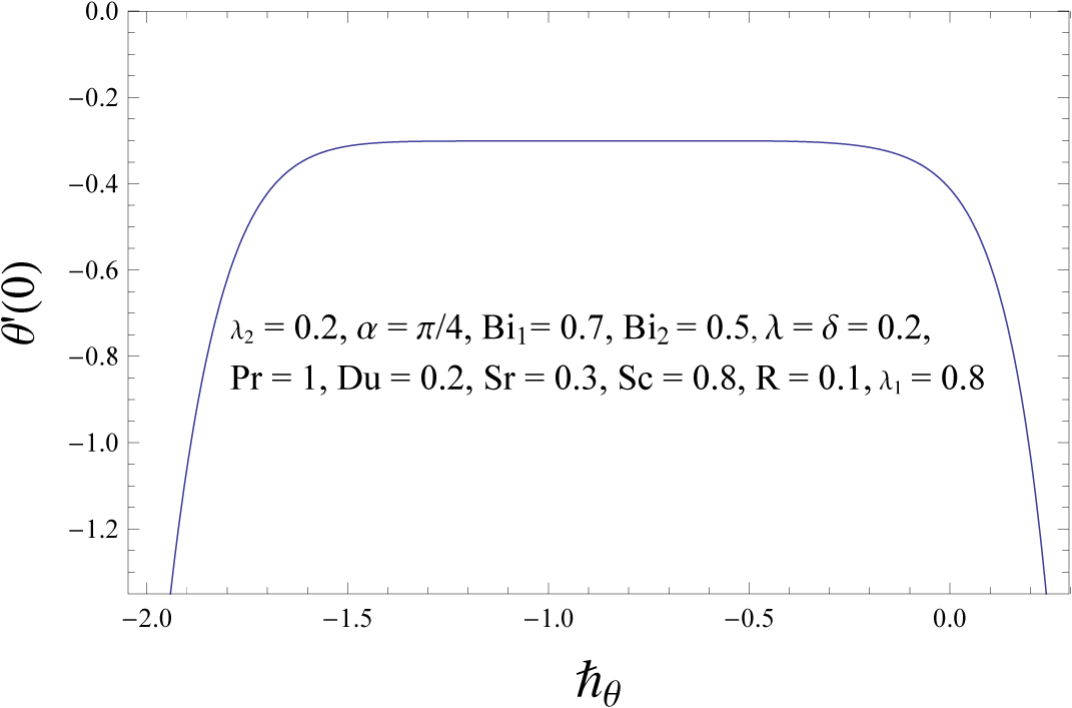
$\hbar_{\theta }$ – curve for temperature field.

**Fig 4:**
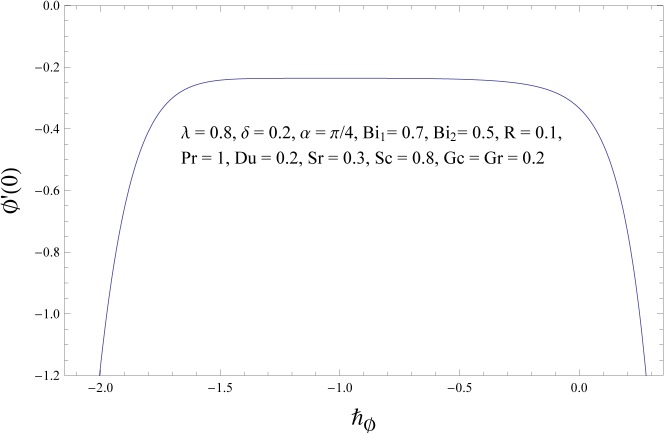
$\hbar_{\phi }$ – curve for concentration field.

Unlike other analytic techniques for nonlinear problems, the homotopy analysis method gives a one-parameter family (in the auxiliary parameter ℏ) of results at any given order of approximations it is the auxiliary parameter ℏ which provides us with a convenient way to adjust and control the convergence of approximations. Any convergent series given by the homotopy analysis method at p = 1 must be one of the exact solutions of considered nonlinear problem. Hence for the given initial guesses and auxiliary parameters, one only needs to choose proper values for ℏ ensuring the series (38–40) converge. To determine the convergence of HAM solution, the ℏ- curve is plotted. Figs 2–4 show that the range of admissible values of $\hbar_{f}$, $\hbar_{\theta }$ and $\hbar_{\phi }$ for some fixed values of parameters are $-1.1\leq \hbar_{f}\leq -0.4$, $-1.3\leq \hbar_{\theta }\leq -0.4$ and $-1.4\leq \hbar_{\phi }\leq -0.5$. The series solutions converge in the whole region of *η* when $\hbar_{f}=-0.8$, $\hbar_{\theta }=-0.8$ and $\hbar_{\phi }=-1.0$. It is obvious from Table 1 that series solutions converge at 25^*th*^ order of approximation.

**Table 1:** Convergence of HAM solutions for different orders of approximations when *λ*_1_ = 0.8, *λ*_2_ = *λ* = *δ* = 0.2, *Sr* = 0.3, *Bi*_1_ = 0.7, *Bi*_2_ = 0.5, *R* = 0.1, *Du* = 0.2, Pr = 1.0, *Sc* = 0.8 and *α* = *π* / 4.

Order of approximations	-*f*″(0)	-*θ*′(0)	-∅′(0)
1	0.936518	0.34653	0.27612
5	0.919520	0.30253	0.23949
10	0.918187	0.30032	0.23607
15	0.918285	0.30049	0.23608
20	0.918294	0.30049	0.23613
25	0.918291	0.30049	0.23613
30	0.918291	0.30049	0.23613
35	0.918291	0.30049	0.23613

## Results and Discussion

**Fig 5:**
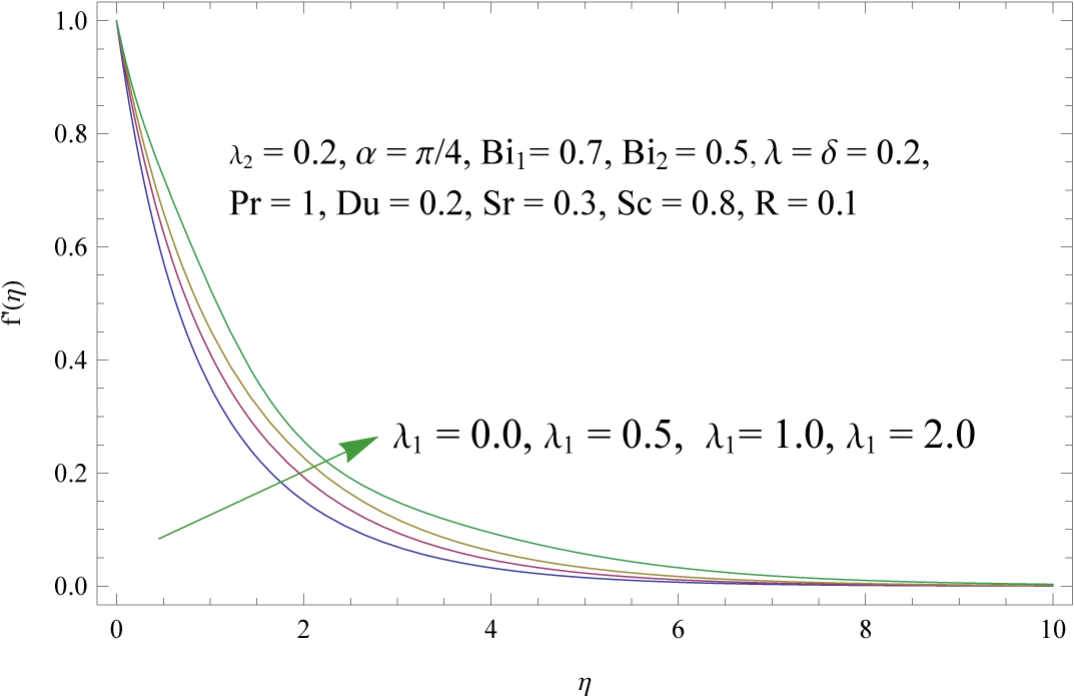
Influence of fluid parameter (*λ*_1_) on *f*′(*η*).

**Fig 6:**
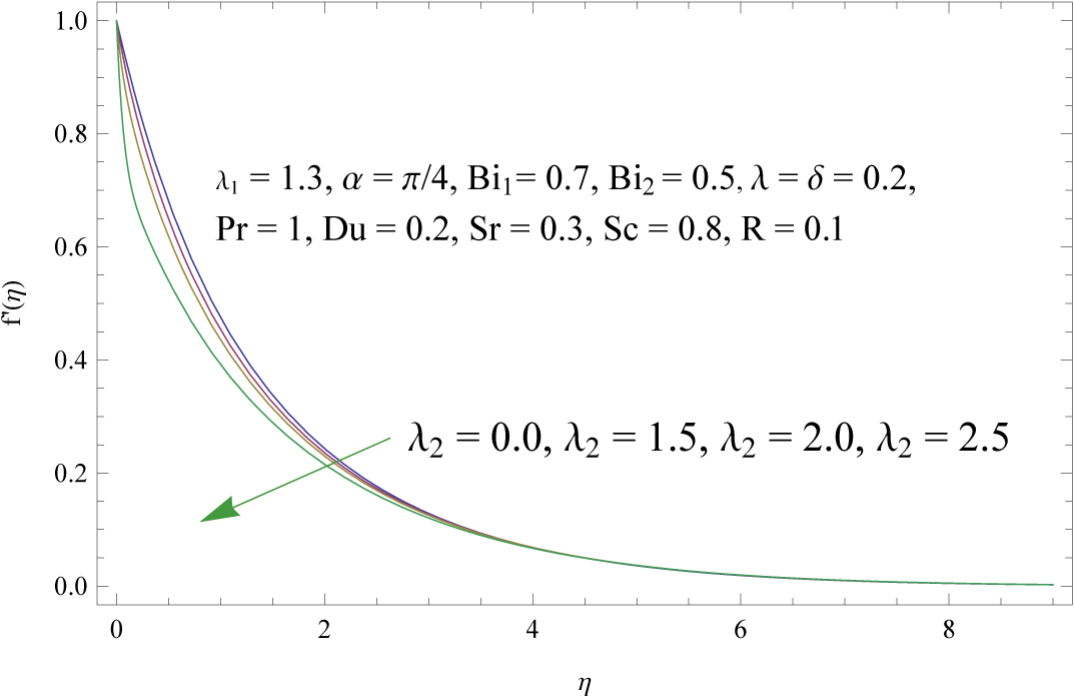
Influence of fluid parameter (*λ*_2_) on *f*′(*η*).

**Fig 7:**
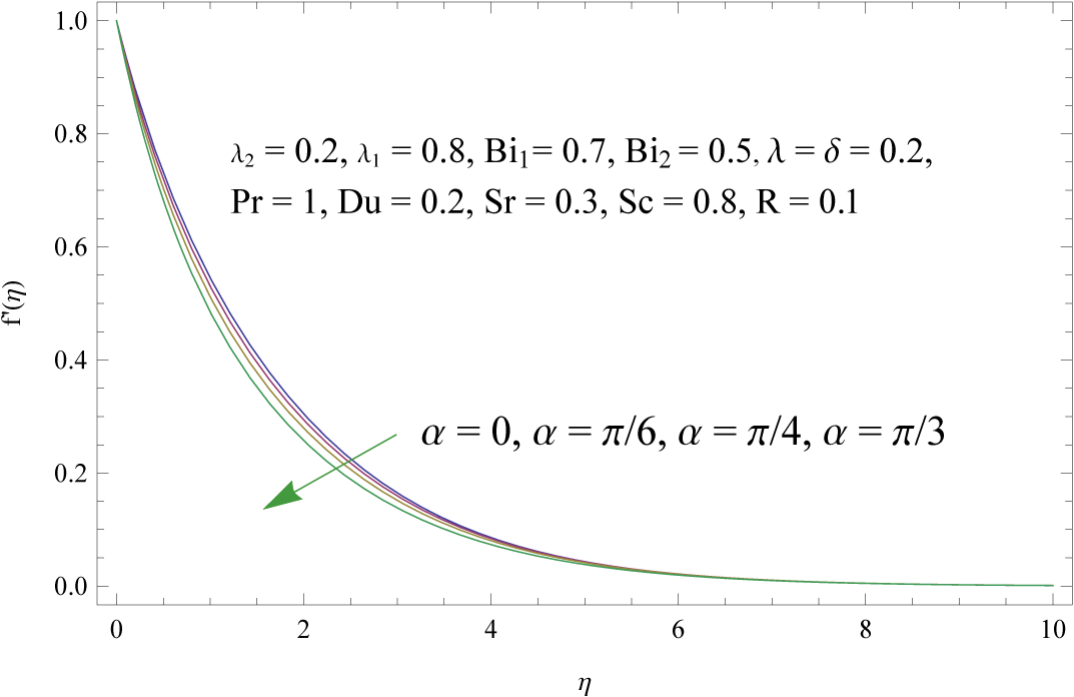
Influence of angle of inclination (*α*) on *f*′(*η*).

**Fig 8:**
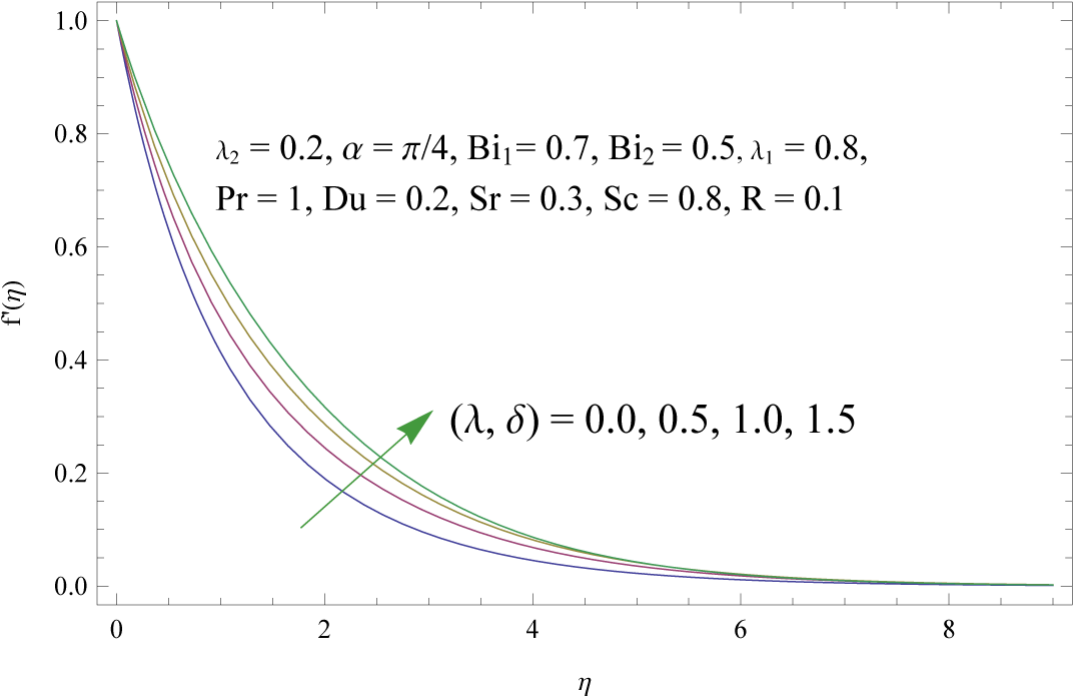
Influences of thermal and solute buoyancy parameters (*λ* and *δ*) on *f*′(*η*).

**Fig 9:**
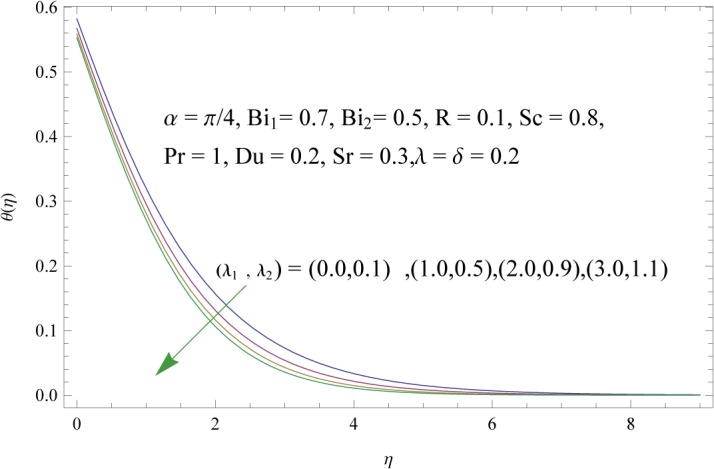
Influences of fluid parameters (*λ*_1_ and *λ*_2_) on *θ*(*η*).

**Fig 10:**
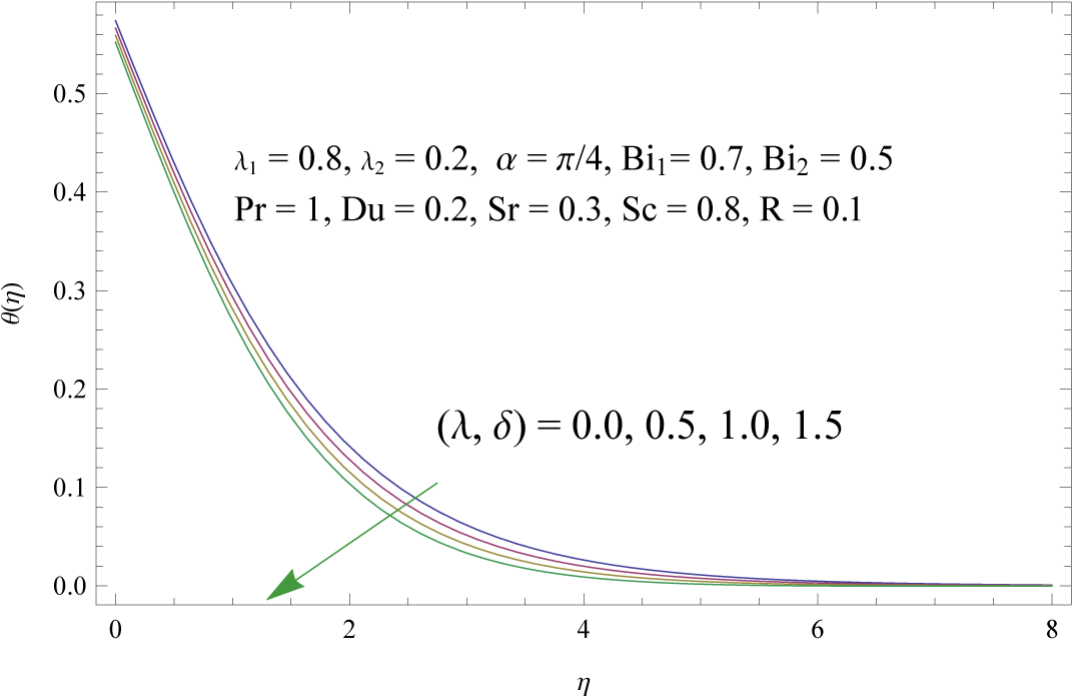
Influences of thermal and solute buoyancy parameters (*λ* and *δ*) on *θ*(*η*).

**Fig 11:**
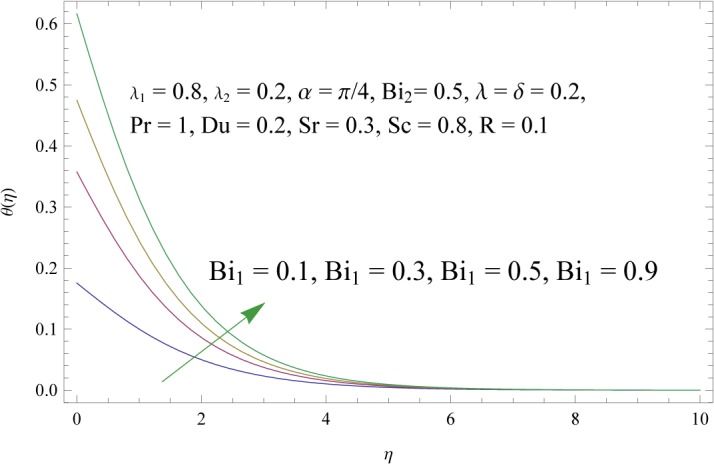
Influence of thermal Biot number (*Bi*_1_) on *θ*(*η*).

**Fig 12:**
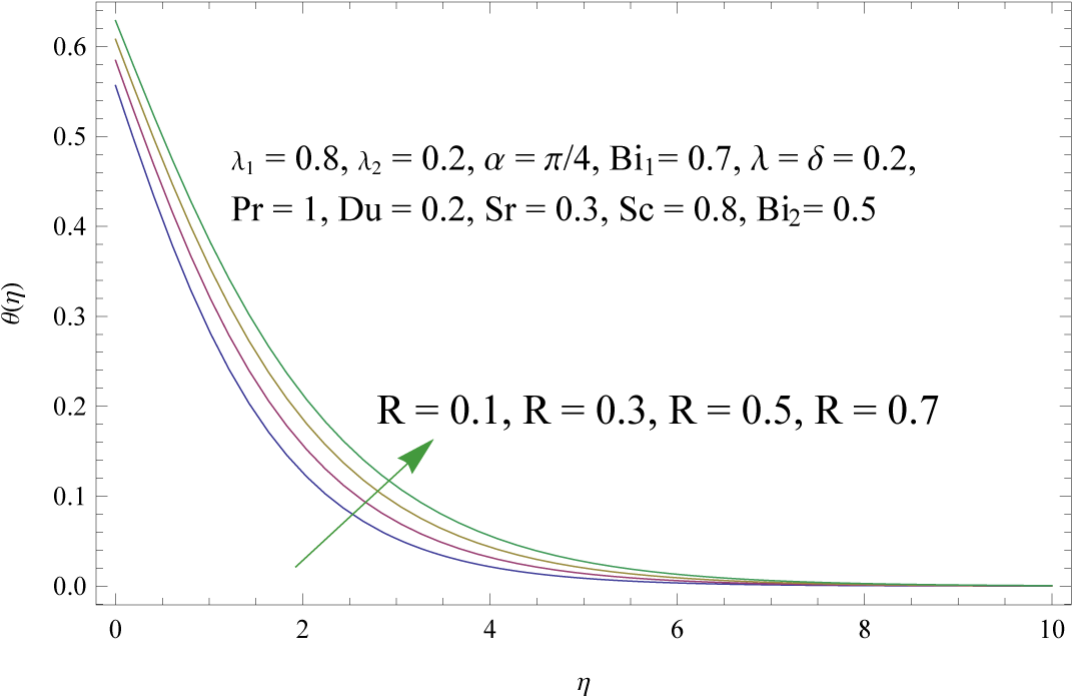
Influence of radiation parameter (*R*) on *θ*(*η*).

**Fig 13:**
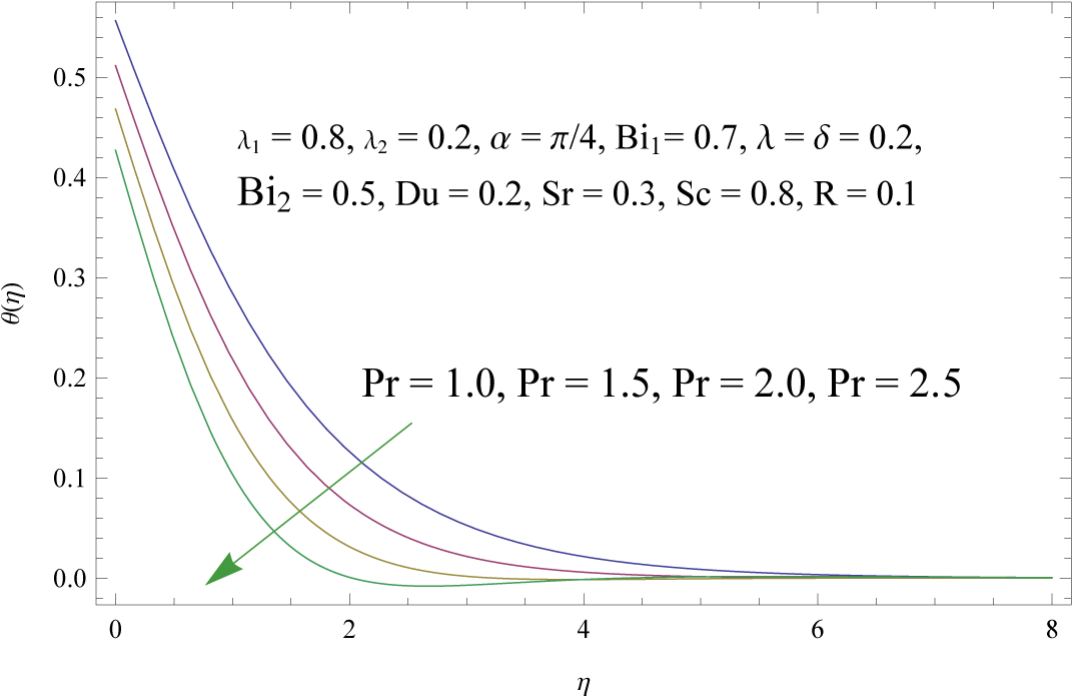
Influence of Prandtl number (Pr) on *θ*(*η*).

**Fig 14:**
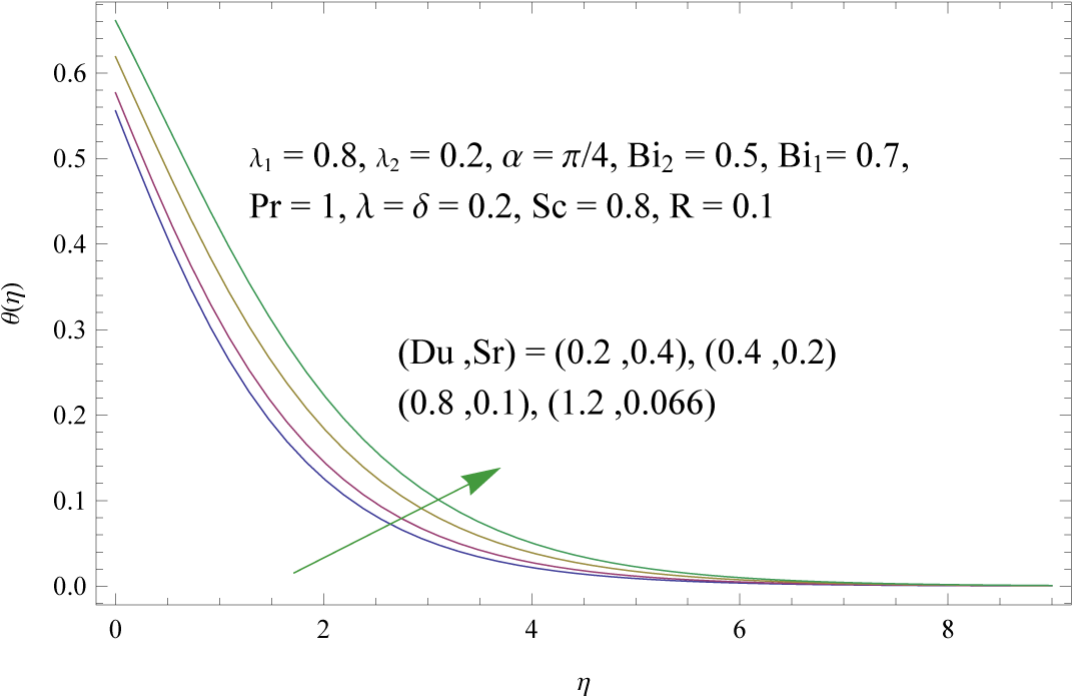
Influences of Dufour and Soret numbers (*Du* and *Sr*) on *θ*(*η*).

**Fig 15:**
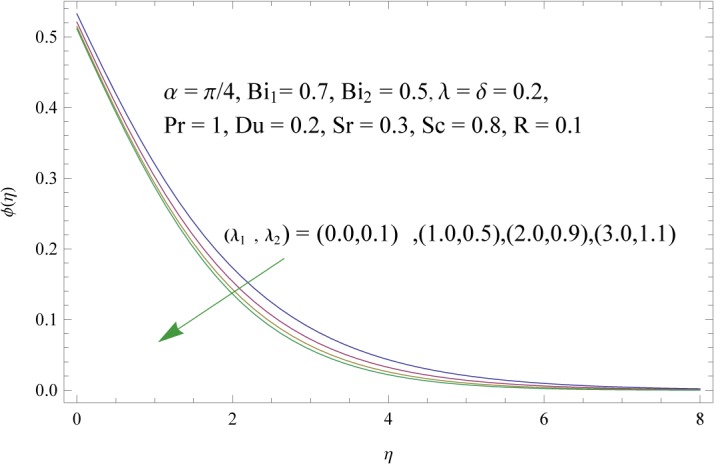
Influences of fluid parameters (*λ*_1_ and *λ*_2_) on *ϕ*(*η*).

**Fig 16:**
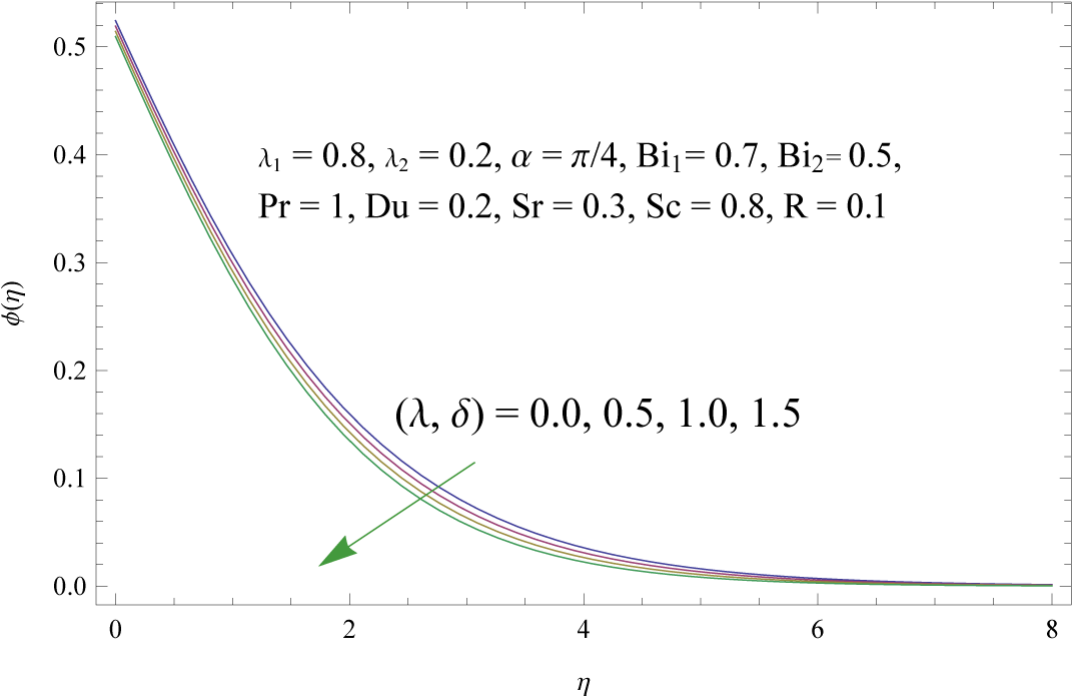
Influences of thermal and solute buoyancy parameters (*λ* and *δ*) on *ϕ*(*η*).

**Fig 17:**
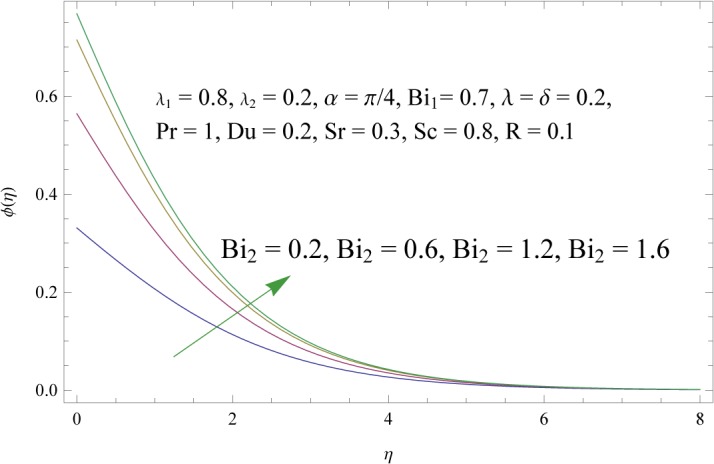
Influence of concentration Biot number (*Bi*_2_) on *ϕ*(*η*).

**Fig 18:**
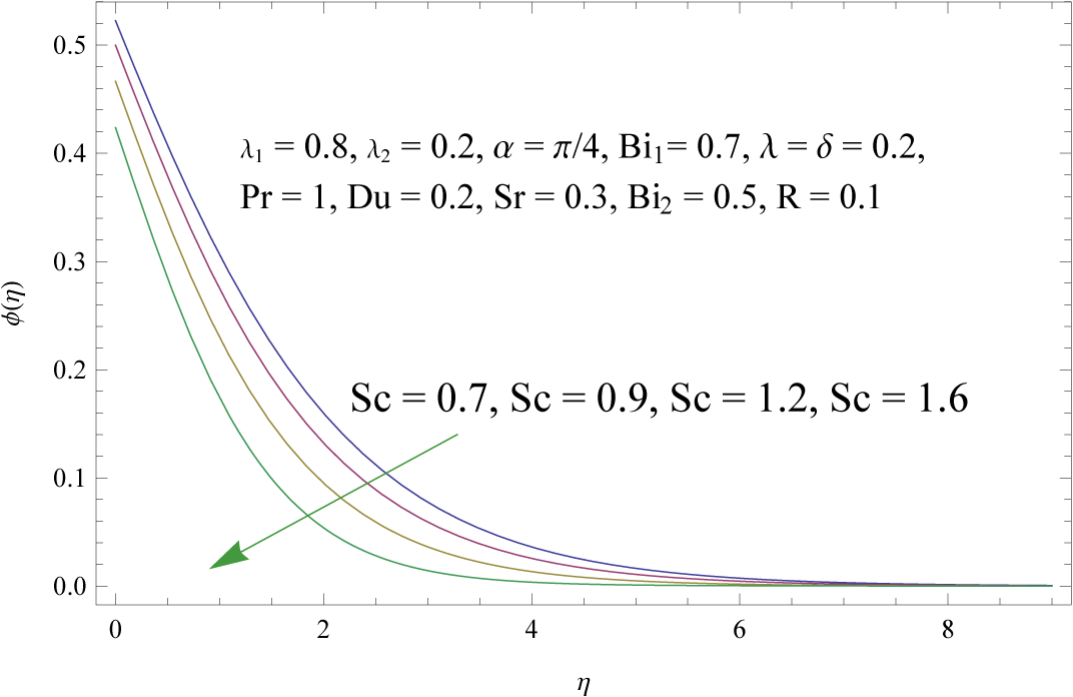
Influence of Schmidt number *Sc* on *ϕ*(*η*).

**Fig 19:**
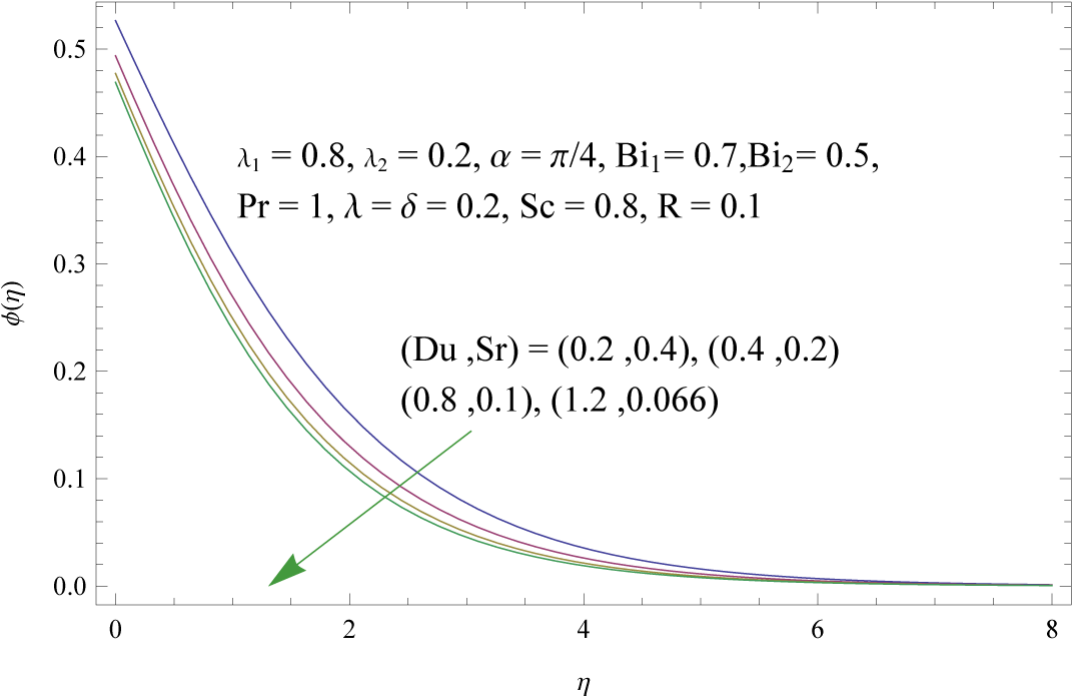
Influences of Dufour and Soret numbers (*Du* and *Sr*) on *ϕ*(*η*).

In order to get a better physical insight of the problem, the dimensionless velocity, temperature and concentration fields are shown graphically. Dimensionless velocity profile *f*′(*η*) is depicted in Figs 5–8 for various values of physical parameters. Influence of fluid parameter *λ*_1_ is shown in Fig 5. By increasing *λ*_1_ the viscosity decreases and hence velocity and momentum boundary layer thickness is increased. Fig 6 presents the effect of fluid parameter *λ*_2_. Increase in *λ*_2_ shows decrease in the velocity and momentum boundary layer thickness. The inclination angle *α* has decreasing impact on the velocity field (see Fig 7). In fact an increase in *α* reduces the buoyancy forces. Combined effects of thermal and solute buoyancy parameters are depicted in Fig 8. By increasing *λ* and *δ* the buoyancy forces increase which enhance the velocity field. Figs 9–15 illustrate the temperature field for different physical parameters involved in problem. Fig 9 displays the variation of temperature profile for various values of *λ*_1_ and *λ*_2_. Larger values of these parameters correspond to the decrease in temperature and thermal boundary layer thickness. Through simultaneous increase of *λ* and *δ* the buoyancy forces are increased. As a result the temperature field is decreased (see Fig 10). Fig 11 shows that a pronounced increase is observed in the temperature and corresponding boundary layer thickness when there is an increase in thermal Biot number *Bi*_1_. Larger values of radiation parameter R have the tendency to enhance the thermal boundary layersee Fig 12. Effect of Prandtl number Pr on the temperature field is plotted in Fig 13. Increase in Prandtl number greatly reduces the temperature and thermal boundary layer. Temperature profile for collective variation of Dufour and Soret numbers is shown in Fig 14. It is noticed that an increase in *Du* (decreasein *Sr*) serves strongly to increase temperature field in the regime. Figs 15–19 illustrate the behavior of concentration field corresponding to involved physical parameters. Effect of fluid parameters (*λ*_1_ and *λ*_2_) is to decrease concentration boundary layer see Fig 15. Increase of *λ* and *δ*, has tendency to decrease the concentration field and associated boundary layer (see Fig 16). Fig 17 indicates that increase of mass Biot number enhances the concentration field. The variation of Schmidt number *Sc* on the concentration field is displayed in Fig 18. Larger values of Schmidt number increase the viscosity and consequently the concentration field is reduced. Combined variation of Dufour and Soret numbers is displayed in Fig 19. Increasing Dufour number *Du* (decreasing Soret number *Sr*) decreases the influence of temperature gradient on the concentration and finally it reduces the concentration field.

Tables 2–4 are prepared to see the values of local skin friction coefficient, local Nusselt and local Sherwood numbers for different embedding parameters involved in the problem. In particular, from Table 2 it is observed that |CfxRex1/2| increases with the increase of *λ*_2_ and *α* while reverse behavior is observed for larger values of *λ*_1_, *λ* and *δ*. It is noticed from Table 3 that Nusselt number decreases for larger values of *λ*_2_ and *α* but it increases by increasing *λ*_1_, *λ*, *δ* and *R*. The variations of Pr, *Du*, *Sr* and *Sc* on the temperature gradient can be seen in Table3. Opposite trend is observed for surface heat transfer coefficient by increasing thermal and concentration Biot numbers (*Bi*_1_ and *Bi*_2_). Local Sherwood numbers are tabulated in Table 4. It is found that the values of local Sherwood number decrease with an increase in *λ*_2_, *Bi*_1_ and *Sr*. It is also observed from the Table that increasing values of *λ*_1_, *Bi*_2_, *Du* and *Sc* cause the increase in mass transfer coefficient. Similar trend is observed for *λ* and *δ* here.

**Table 2:** Values of skin-friction coefficient CfxRex1/2 for different parameters.

*λ*_1_	*λ*_2_	α	δ	*λ*	−*Cf*_*x*_ *Re*_*x*_^1/2^
0.8	0.2	*π*/4	0.2	0.2	0.917213
0.5					0.998027
0.8					0.917213
1.0					0.872685
	0.0				0.900719
	0.1				0.909740
	0.25				0.921701
		*π*/6			0.904726
		*π*/4			0.917213
		*π*/3			0.933805
			0.1		0.931610
			0.3		0.903116
			0.4		0.889291
				0.3	0.931095
				0.4	0.903555
				0.5	0.890106

**Table 3:** Values of local Nusselt number Nux/Rex1/2 for different parameters.

λ_1_	λ_2_	δ	λ	α	R	Pr	Du	Sr	Sc	*Bi*_1_	*Bi*_2_	*Nu*_*x*_ */Re*_*x*_^1/2^
0.3	0.8	0.2	0.2	π/4	0.1	1.0	0.2	0.3	0.8	0.7	0.5	0.33581
0.4												0.34055
0.5												0.34322
	0.5											0.34035
	0.8											0.34015
	1.0											0.33993
		0.3										0.34161
		0.4										0.34261
		0.5										0.34357
			0.3									0.34153
			0.4									0.34314
			0.5									0.34336
				π/6								0.34147
				π/4								0.34055
				π/3								0.33929
					0.1							0.34055
					0.2							0.36593
					0.3							0.38986
						1.1						0.35180
						1.2						0.36206
						1.3						0.37145
							0.1					0.34840
							0.3					0.33263
							0.4					0.32465
								0.3				0.34055
								0.4				0.34148
								0.5				0.34242
									0.7			0.34173
									0.9			0.33953
									1.0			0.33862
										0.5		0.28813
										0.6		0.31655
										0.8		0.36110
											0.5	0.34055
											0.6	0.33914
											0.8	0.33695

**Table 4:** Values of local Sherwood number Shx/Rex1/2 for different parameters.

λ	λ	λ	δ	Sc	Sr	Du	*Bi*_1_	*Bi*_2_	*Sh*_*x*_ */Re*_*x*_^1/2^
0.5	0.2	0.2	0.2	0.8	0.3	0.2	0.7	0.5	0.23278
0.8									0.23613
1.0									0.23802
	0.1								0.23626
	0.3								0.23600
	0.4								0.23586
		0.1							0.23540
		0.3							0.23683
		0.4							0.23749
			0.1						0.23532
			0.3						0.23689
			0.4						0.23760
				0.7					0.22663
				0.9					0.24439
				1.0					0.25166
					0.2				0.24358
					0.3				0.23613
					0.5				0.22110
						0.1			0.23533
						0.3			0.23694
						0.4			0.23775
							0.5		0.23941
							0.6		0.23763
							0.8		0.23484
								0.4	0.20882
								0.6	0.25870
								0.8	0.29386

## Conclusions

Simultaneous effects of convective heat and mass transfer in the flow of Powell-Erying fluid past an inclined exponential stretching surface with Soret and Dufour effects are investigated in this article. The following points of performed analysis are worthmentioning.

- The velocity field has opposite results for both the fluid parameters *λ*_1_ and *λ*_2_.
- Inclination angle *α* reduces the velocity and momentum boundary layer.
- The temperature and concentration are decreased by increasing values of fluid parameters *λ*_1_ and *λ*_2_.
- Variation of thermal and solute buoyancy parameters on the temperature and concentration fields is reverse to that of velocity.
- Prandtl number has remarkable effect on the temperature while dual behavior is observed for concentration field.
- The behaviors of thermal and mass Biot numbers corresponding to temperature and concentration are quite similar.
- Qualitatively opposite behavior is observed for temperature and concentration profiles for Soret and Dufour numbers.
- A concentration profile is decreasing function of *Sc*.
- As *Bi*_1_, *Bi*_2_ → ∞, the convective boundary conditions are reduced to limiting case of prescribed surface temperature and concentration respectively.
- When fluid parameters *λ*_1_ and *λ*_2_ → 0, the present problem reduces to viscous case.

## References

[1] Crane LJ. Flow past a stretching plate. J. Appl. Math. Phy. 1970; 21: 645–647.

[2] Hayat T, Mustafa M, Pop I. Heat and mass transfer for Soret and Dufour's effect on mixed convection boundary layer flow over a stretching vertical surface in a porous medium filled with a viscoelastic fluid. Comm. Nonlinear Sci. Numer. Simul. 2010; 15: 1183–1196.

[3] Das K. Effect of chemical reaction and thermal radiation on heat and mass transfer flow of MHD micropolar fluid in a rotating frame of reference. Int. J. Heat Mass Transfer. 2011; 54: 3505–3513.

[4] Joneidi AA, Domairry G, Babaelahi M. Analytical treatment of MHD free convective flow and mass transfer over a stretching sheet with chemical reaction. J. Taiwan Inst. Chem. Eng. 2010; 41: 35–43.

[5] Hayat T, Qasim M, Abbas Z. Homotopy solution for the unsteady three-dimensional MHD flow and mass transfer in a porous space. Comm. Nonlinear Sci. Numer. Simul. 2010; 15: 2375–2387.

[6] Khani F, Aziz A, Hamedi-Nezhad S. Simultaneous heat and mass transfer in natural convection about an isothermal vertical plate. J. King Saud University-Sci. 2012; 24: 123–129.

[7] Kandasamy R, Hayat T, Obaidat S. Group theory transformation for Soret and Dufour effects on free convective heat and mass transfer with thermophoresis and chemical reaction over a porous stretching surface in the presence of heat source/sink. Nuclear Eng. Design, 241 2011; 21: 55–2161.

[8] Pal D, Chatterjee S. Soret and Dufour effects on MHD convection heat and mass transfer of a power-law fluid over an inclined plate with variable thermal conductivity in a porous medium. Appl. Math. Comput. 2013; 219: 7556–7574.

[9] Auranzaib, Shafie S. Thermal diffusion and diffusion thermo effects on unsteady MHD free convection flow over a stretching surface considering joule heating and viscous dissipation with thermal stratification, chemical reaction and Hall current. J. Franklin Inst. 2013; 351: 1268–1287.

[10] Turkyilmazoglu M. Multiple solutions of heat and mass transfer of MHD slip flow for the viscoelastic fluid over a stretching sheet. Int. J. Thermal Sci. 2011; 50: 2264–2276.

[11] Aziz A. A similarity solution for laminar thermal boundary layer over a flat plate with a convective surface boundary condition. Comm. Nonlinear Sci. Numerical Simul. 2009; 14: 1064–1068.

[12] Patil PM, Momoniat E, Roy S. Influence of convective boundary condition on double diffusive mixed convection from a permeable vertical surface. Int. J. Heat Mass Transfer. 2014; 70: 313–321.

[13] Ramesh GK, Gireesha BJ. Influence of heat source/sink on a Maxwell fluid over a stretching surface with convective boundary condition in the presence of nanoparticles. Ain Shams Eng. J. 2014; 5: 991–998.

[14] Rashad AM, Chamkha AJ, Modather M. Mixed convection boundary-layer flow past a horizontal circular cylinder embedded in a porous medium filled with a nanofluid under convective boundary condition. Computers ^& Fluids. 2013; 86: 380–388.

[15] Alsaedi A, Awais M, Hayat T. Effects of heat generation/absorption on stagnation point flow of nanofluid over a surface with convective boundary conditions. Comm. Nonlinear Sci. Numer. Simul. 2012; 17: 4210–4223.

[16] Hamad MAA, Uddin Md J, Ismail AI-Md. Investigation of combined heat and mass transfer by Lie group analysis with variable diffusivity taking into account hydrodynamic slip and thermal convective boundary conditions. Int. J. Heat Mass Transfer. 2012; 55: 1355–1362.

[17] Makinde OD, Aziz A. Boundary layer flow of a nanofluid past a stretching sheet with a convective boundary condition. Int. J. Thermal Sci. 2011; 50: 1326–1332.

[18] Shehzad SA, Alsaedi A, Hayat T. Three-dimensional flow of Jeffery fluid with convective surface boundary conditions. Int. J. Heat Mass Transfer. 2012; 55: 3971–3976.

[19] Das K, Duari PR, Kundu PK. Numerical simulation of nanofluid flow with convective boundary condition. J. Egyptian Math. Soc. 2014; 6 27.

[20] Hayat T, Iqbal Z, Mustafa M, Alsaedi A. Momentum and heat transfer of an upper-convected Maxwell fluid over a moving surface with convective boundary conditions. Nuclear Eng. Design, 2012; 252: 242–247.

[21] Liao S. An optimal homotopy-analysis approach for strongly nonlinear differential equations. Comm. Nonlinear Sci. Numer. Simul. 2010; 15: 2003–2016.

[22] Abbasbandy S, Hashemi MS, Hashim I. On convergence of homotopy analysis method and its application to fractional integro-differential equations. Quaestiones Math. 2013; 36: 93–105.

[23] Rashidi MM, Rostami B, Freidoonimehr N Abbasbandy S. Free convective heat and mass transfer for MHD fluid flow over a permeable vertical stretching sheet in the presence of the radiation and buoyancy effects. Ain Shams Eng. J. 2014; 5: 901–912.

[24] Awais M, Hayat T, Alsaedi A, Asghar S. Time-dependent three-dimensional boundary layer flow of a Maxwell fluid. Computer & Fluids. 2014; 91: 21–27.

[25] Rostami B, Rashidi MM, Rostami P, Momoniat E, Freidoonimehr N. Analytical investigation of laminar viscoelastic fluid flow over a wedge in the presence of buoyancy force effects. Abstract Appl. Analy., 2014: 2014; ID 496254.

[26] Sheikholeslami M, Ellahi R, Ashorynejad HR, Domairry G, Hayat T. Effect of heat transfer in flow of nanofluids over a permeable stretching wall in a porous medium. J. Comput. Theor. Nanosci. 2014; 11: 486–496.

[27] Turkyilmazoglu M. Effective computation of exact and analytic approximate solutions to singular nonlinear equations of Lane-Emden-Flower type. Appl. Math. Model. 2013; 37: 7539–7548.

[28] Turkyilmazoglu M. Effective computation of solutions for nonlinear heat transfer problems in fins. J. Heat Transfer. 2014; 136: 091901.

[29] Hayat T, Asad S, Alsaedi A. Flow of variable thermal conductivity fluid due to inclined stretching cylinder with viscous dissipation and thermal radiation. Appl. Math. Mech. 2014; 35: 1–12.

[30] Hayat T, Asad S, Mustafa M, Alsulami HH. Heat transfer analysis in flow of Walters' B fluid with convective boundary condition. Chin. Phy. B, 2014; 23: 084701.

